# Beyond BMI: insulin resistance emerges as the key metabolic correlate of AMH in PCOS

**DOI:** 10.3389/fendo.2026.1892970

**Published:** 2026-07-09

**Authors:** Amalia Gorzko, Jolanta Nawrocka-Rutkowska, Edyta Śliwak, Andrzej Starczewski, Iwona Szydłowska

**Affiliations:** Department of Gynecology, Endocrinology and Gynecological Oncology, Pomeranian Medical University in Szczecin, Szczecin, Poland

**Keywords:** AMH, BMI, fasting insulin, HOMA-IR, PCOS

## Abstract

**Background:**

The association between anti-Müllerian hormone (AMH) levels, body weight and insulin resistance in women with polycystic ovary syndrome (PCOS) has been extensively discussed, yet evidence remains conflicting. Therefore, the aim of this study was to determine the relationship between AMH levels and metabolic parameters among reproductive-age women with PCOS in Poland.

**Methods:**

This retrospective study included 156 women diagnosed with PCOS according to the Rotterdam criteria and 156 controls without PCOS. Serum AMH, fasting glucose, and fasting insulin concentrations were measured, and insulin resistance was assessed using the homeostasis model assessment (HOMA-IR). Multiple regression, interaction, and mediation analyses were performed to evaluate the relationships between BMI, insulin resistance, and AMH levels.

**Results:**

A negative association between AMH concentration and both fasting insulin and HOMA-IR was revealed. Subsequent analysis further demonstrated that the relationship between HOMA-IR and AMH is independent of BMI, whereas the association between BMI and AMH was weaker and likely mediated by insulin resistance.

**Conclusions:**

Insulin resistance appears to represent a stronger metabolic correlate of AMH levels than BMI in women with PCOS. These findings may suggest that metabolic dysfunction, rather than excess body weight alone, is more closely associated with AMH alterations in PCOS.

## 1 Introduction

Polycystic ovary syndrome (PCOS) is one of the most common endocrine disorders in women of reproductive age, affecting approximately 10–15% of this population (1). The syndrome is characterized by marked heterogeneity and a strong metabolic component, including insulin resistance, compensatory hyperinsulinemia, and an increased risk of metabolic syndrome, cardiovascular disease, and type 2 diabetes mellitus (2). In this context, recent consensus discussions have proposed a re-conceptualization of PCOS as polyendocrine metabolic ovarian syndrome (PMOS) to better reflect its systemic metabolic–endocrine nature; however, PCOS remains the established terminology in current literature (3).

Anti-Müllerian hormone (AMH), a glycoprotein of the transforming growth factor beta (TGF-β) family secreted by granulosa cells of preantral and small antral follicles, is widely used as a marker of ovarian reserve, reflecting the number and developmental stage of growing follicles (4). Serum AMH levels decline with age and may be influenced by factors such as hormonal contraceptive use, smoking status, and ethnicity (5, 6). Emerging evidence suggests that AMH may also be modulated by metabolic status in PCOS, although findings remain inconsistent across populations, particularly regarding its relationship with adiposity and insulin resistance (6, 7).

Insulin resistance (IR) is a common metabolic feature in PCOS, affecting up to 70% of women with obesity and approximately 30% of lean individuals (8). It is characterized by reduced insulin sensitivity in peripheral tissues, including skeletal muscle, liver, and adipose tissue, and results from both genetic and environmental factors such as physical inactivity, diet, and medication use (8). In PCOS, IR is mainly driven by post-receptor defects in insulin signaling, which impair glucose transport and are closely associated with hyperandrogenism (9, 10). A widely used surrogate marker of IR is the Homeostasis Model Assessment of Insulin Resistance (HOMA-IR), with values >2.5 indicating insulin resistance in adults (9). IR is strongly associated with central obesity, which promotes insulin resistance through inflammatory mediators and increased free fatty acids, further exacerbating metabolic and endocrine dysfunction in PCOS (9, 10). Management includes lifestyle interventions and pharmacological treatment such as metformin (1, 9–11).

Given the close interplay between insulin resistance and reproductive endocrine dysfunction in PCOS, the relationship between IR and AMH has emerged as a clinically relevant but insufficiently understood area of investigation. Therefore, this study aims to examine the association between insulin resistance and AMH levels in a cohort of Eastern European women with PCOS, addressing an underrepresented population in current literature.

## 2 Patients and methods

### 2.1 Study group:

This retrospective study involved women aged 18–49 years from the Polish population. Prior to enrollment, participants were fully informed about the study objectives and procedures, provided written informed consent, and authorized the processing of their personal data in accordance with the European Union General Data Protection Regulation (Official Journal of the European Union L 119, 4 May 2016, p. 1)). The study protocol received approval from the Bioethics Committee of the Pomeranian Medical University (approval no. KB-006/12/2024).

The study group consisted of 156 women diagnosed with PCOS according to the Rotterdam criteria, while a control group included 156 women who did not meet these diagnostic criteria. Detailed medical histories were obtained from all participants, and individuals with systemic conditions that could potentially influence study outcomes (such as diabetes, thyroid dysfunction, hypertension, cardiovascular, or hepatic disorders) were excluded. Any ongoing hormonal therapies were discontinued at least three months prior to participation.

All participants underwent gynecological examination and transvaginal ultrasound to assess ovarian volume and follicular characteristics using an Alpinion X-CUBE 70 device equipped with an 11 MHz endovaginal probe. Blood samples were collected to measure serum AMH, fasting glucose, and fasting insulin levels. Insulin resistance was evaluated using the HOMA-IR index, with a cutoff value of >2.5 indicating insulin resistance. This threshold was selected because it is commonly used in adult populations and in studies evaluating metabolic disturbances in women with PCOS, although no universally accepted cutoff value exists (12). Body weight and height were measured to calculate body mass index (BMI, kg/m²), which was subsequently categorized according to WHO standards as underweight, normal weight, overweight, or obese (9).

### 2.2 Biochemical tests:

The serum samples (5 mL of venous blood) were obtained on days 3-5 (early follicular phase) of the menstrual cycle. To ensure the validity of the results, all laboratory tests were performed in the same laboratory. Measurements were performed using Roche kits, with AMH levels assessed by the Elecsys assay via the ECLIA method on a Cobas analyzer (Roche Diagnostics, Mannheim, Germany). Glucose concentrations were measured using an enzymatic hexokinase assay on a Cobas analyzer, whereas insulin levels were determined by electrochemiluminescence immunoassay (Roche Diagnostics, Mannheim, Germany) performed on a Cobas e system.

### 2.3 Statistical analysis

The normality of distributions was tested using the Kolmogorov-Smirnov and Shapiro-Wilk tests. Spearman’s rank correlations, multiple and partial correlations were calculated in STATISTICA 13 (TIBCO Software Inc.). The dependent variable was anti-Müllerian hormone after natural-log transformation (lnAMH). Predictors included body mass index (BMI) and insulin-related markers: fasting insulin (natural log; lnINS) and/or the homeostatic model assessment of insulin resistance (natural log; lnHOMA). Spearman’s rank correlation coefficients were calculated to assess bivariate associations between AMH and metabolic parameters, including BMI, fasting glucose, fasting insulin, and HOMA-IR. Multiple linear regression models with interaction terms (BMI × lnINS/lnHOMA; predictors were mean-centered) and assessed significance using Type II ANOVA were fitted. The mediation hypothesis (BMI → lnINS/lnHOMA → lnAMH) were additionally tested using causal mediation analysis with a nonparametric bootstrap (5,000 replicates), reporting Average Causal Mediation Effect (ACME -indirect effect), Average Direct Effect (ADE), total effect, and the proportion mediated. Results are presented as standardized coefficients and with percentage interpretations appropriate for log-linear models. Analyses were performed in R 4.5.0 (Windows 11) using the mediation, dplyr/readr, car, interactions, and ggplot2 packages.

The significance level was set at p<0.05. A statistical tendency was described in cases where the p-value was close to 0.05.

## 3 Results

The characteristics of the study and control groups are presented in **Table 1**. The study and control groups do not differ statistically in age or fasting glucose concentration. The study group is characterized by significantly higher values of BMI, AMH, insulin levels, as well as HOMA-IR (p<0.005). The difference between the study group and the control group in the logarithmized values is statistically significant only for lnAMH; in the case of insulin concentration, it appears only as a statistical trend (0.10 > p > 0.05). It is not statistically significant for lnglucose and lnHOMA-IR.

**Table 1:** Characteristics of the groups.

Parameters		PCOS group	Non- PCOS group	P value
age [years]	mean ± SD	26.564 ± 5.229	27.590 ± 6.938	NS*
BMI [kg/m2]	mean ± SD	28.159 ± 5.895	25.347 ± 5.408	p<0.001
AMH [ng/ml]	mean ± SD	7.831 ± 4.490	3.496 ± 2.563	p<0.001
	lnAMH^1^ mean ± SD	1.918 ± 0.536	0.999 ± 0.752	p<0.001
fasting glucose [mg/dl]	mean ± SD	92.558 ± 8.402	91.328 ± 7.991	NS
	lnglucose^1^ mean ± SD	4.524 ± 0.088	4.511 ± 0.085	NS
insulin [mU/ml]	mean ± SD	15.564 ± 13.435	12.721 ± 8.483	p<0.05
	lniINS^1^ mean ± SD	2.485 ± 0.705	2.351 ± 0.628	0.10<p>0.05
HOMA-IR	mean ± SD	3.653 ± 3.450	2.968 ± 2.032	p<0.05
	lnHOMA-IR^1^ mean ± SD	0.998 ± 0.749	0.880 ± 0.657	NS

* NS, not statistically significant;.

^1^outcomes transformed by natural logarithm;.

All tests made with independent samples t-test.

Spearman’s rank correlation analysis was performed to assess the associations between AMH and metabolic parameters in both groups (**Table 2**). In women with PCOS, AMH was negatively correlated with BMI (ρ = −0.262, p < 0.001), fasting insulin (ρ = −0.298, p < 0.001), and HOMA-IR (ρ = −0.286, p < 0.001). No significant correlation was observed between AMH and fasting glucose (ρ = −0.054, p = 0.504). In the non-PCOS group, AMH was not significantly correlated with BMI, fasting glucose, fasting insulin, or HOMA-IR. These findings indicate that the negative association between AMH and insulin resistance-related parameters was observed only in women with PCOS.

**Table 2:** Spearman rank correlations between AMH and metabolic parameters in PCOS and non-PCOS groups.

Parameter	PCOS group: Spearman’s ρ with AMH	p value	Non-PCOS group: Spearman’s ρ with AMH	p value
BMI	−0.262	<0.001	−0.045	0.581
Fasting glucose	−0.054	0.504	−0.105	0.193
Fasting insulin	−0.298	<0.001	−0.018	0.822
HOMA-IR	−0.286	<0.001	−0.045	0.574

**Figure 1:**
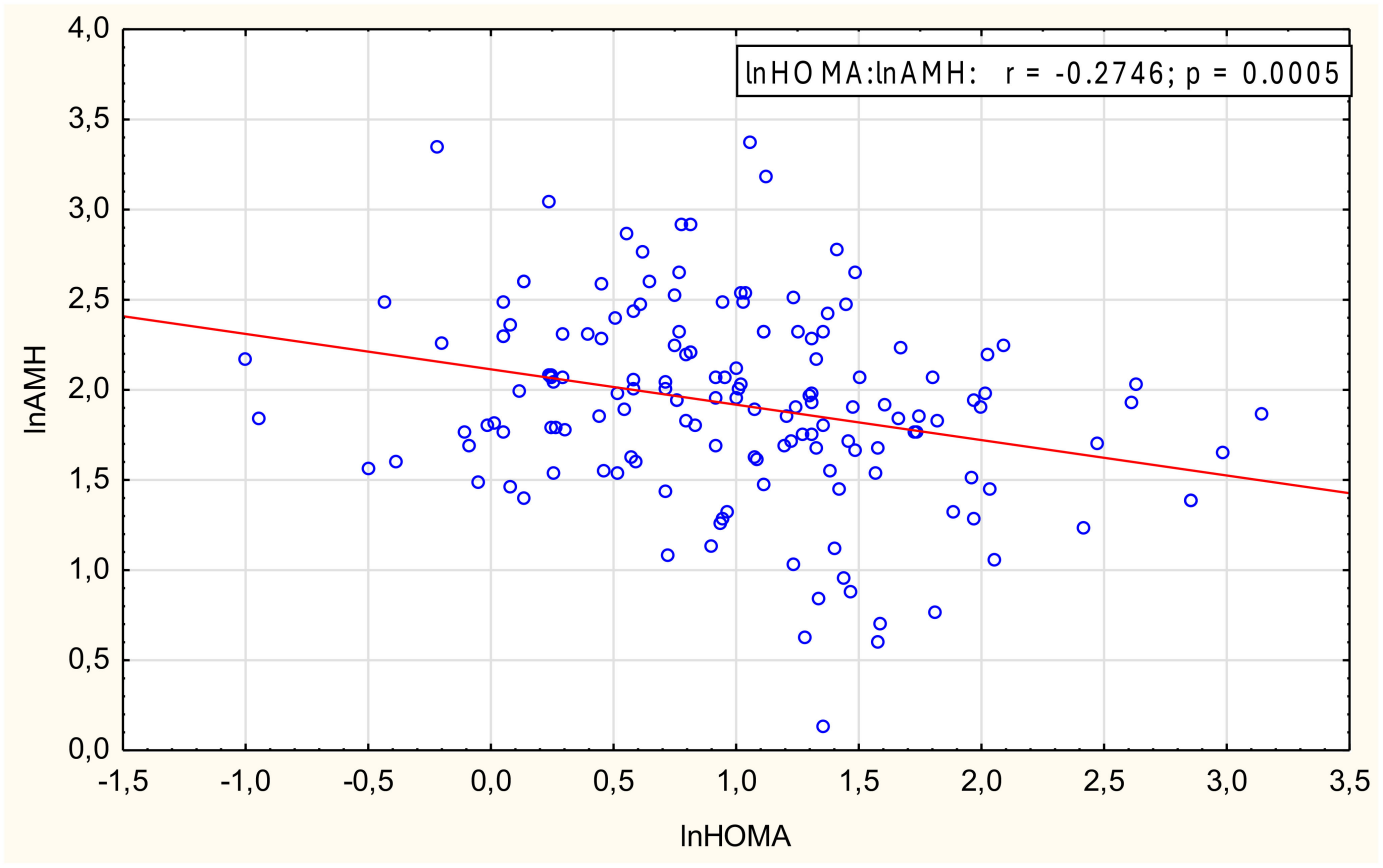
Scatter plot; lnHOMA vs. lnAMH in PCOS women.

**Figure 2:**
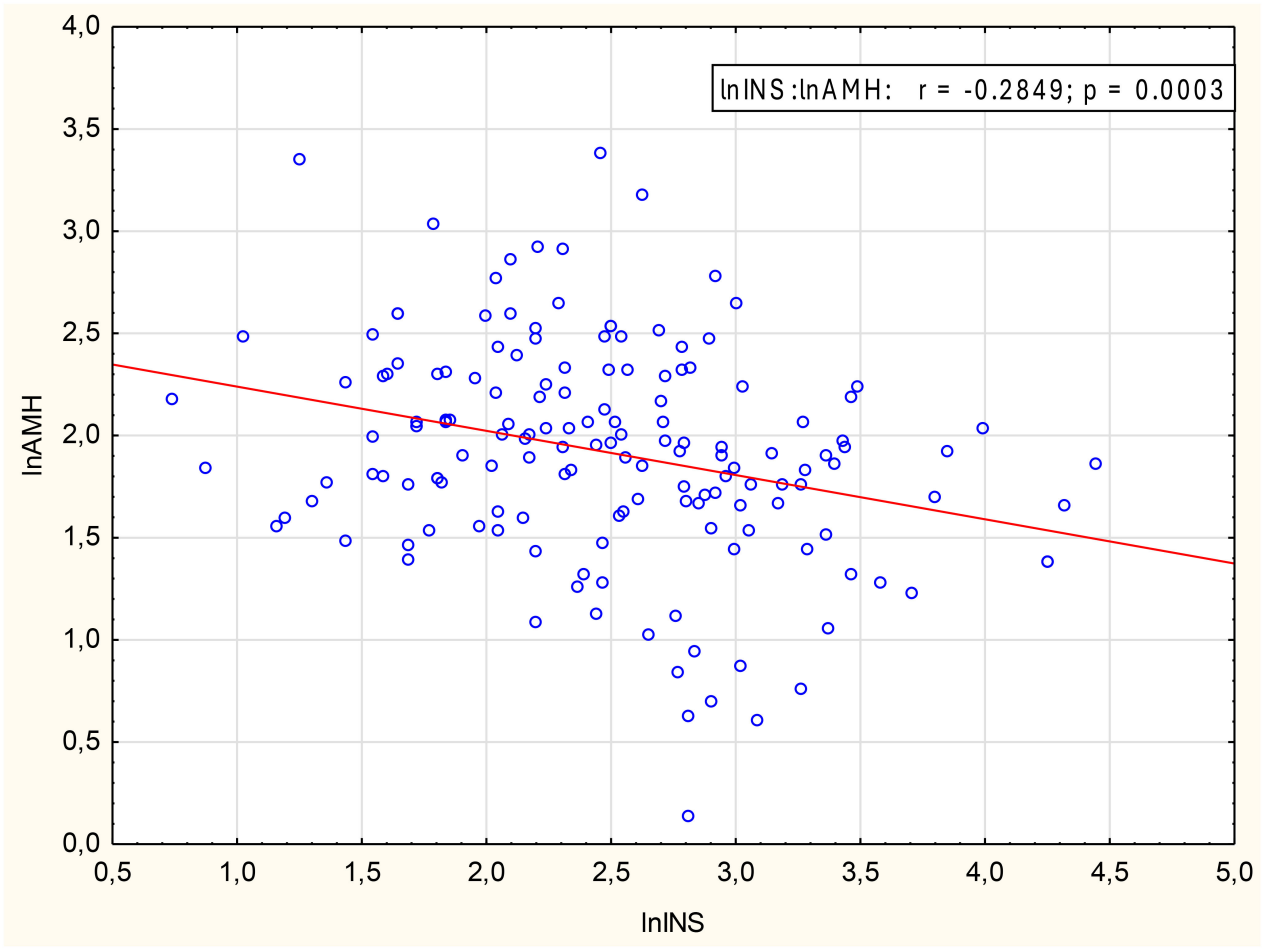
Scatter plot; lnINS vs. lnAMH in PCOS women.

Scatter plots illustrating the associations between lnAMH and lnHOMA-IR as well as between lnAMH and lnINS in women with PCOS are presented in **Figures 1** and **2**. Subsequently, multiple and partial correlation analyses were performed to assess whether the association between insulin resistance and AMH remained evident after accounting for BMI.

The described negative association was not observed between fasting glucose and AMH in PCOS group (r = -0.0958; p = 0.2342).

In the control group, no correlations were observed (lnAMH vs. lnHOMA: r = -0.0515; p = 0.5235; lnAMH vs. lnINS: r = -0.0185; p = 0.8187; lnAMH vs. lnglucose: r = -0.1491; p = 0.0632).

Multiple and partial correlation analyses between AMH (dependent variable), BMI and HOMA-IR (independent variables) demonstrated differential patterns of association in the study and control groups. In the study group, the regression model was statistically significant (F(2.153) = 6.87; p = 0.001), accounting for approximately 8% of the variance in AMH levels (R² = 0.082; adjusted R² = 0.070). Higher lnHOMA values were significantly associated with lower lnAMH concentrations (β = –0.21), whereas BMI showed a weaker negative effect (β = –0.11) with limited statistical significance. The multiple regression model was expressed as:


ln(AMH)=2.34−0.15·ln(HOMA)−0.01·BMI

(SE of estimate=0.52)

In contrast, in the control group the model was not statistically significant (F(2.153) = 0.25; p = 0.78), and the proportion of explained variance was negligible (R² = 0.003; adjusted R² = –0.010). Neither BMI (β = –0.03) nor lnHOMA (β = –0.03) emerged as significant predictors of lnAMH.

To precisely characterize the relationships between BMI, HOMA-IR, and AMH, an interaction analysis between the independent variables was conducted in women with PCOS. Multiple regression analysis including the interaction term (BMI × lnHOMA) showed that the model significantly predicted lnAMH levels in women with PCOS (F(3.152) = 4.58; p = 0.004; R² = 0.083; adjusted R² = 0.065). Among the predictors, only lnHOMA was significant (β = –0.15; p = 0.040), indicating that higher insulin resistance was associated with lower lnAMH values. The effect of BMI did not reach statistical significance (β = –0.01; p = 0.266), and the BMI × lnHOMA interaction was also non-significant (β = 0.002; p = 0.865). Simple slope analysis confirmed the absence of a significant effect of BMI on lnAMH at low, medium, and high levels of lnHOMA.

Subsequently, hypotheses were tested regarding the influence of one of the independent variables described above on the dependent variable through the other independent variable.

**Figure 3:**
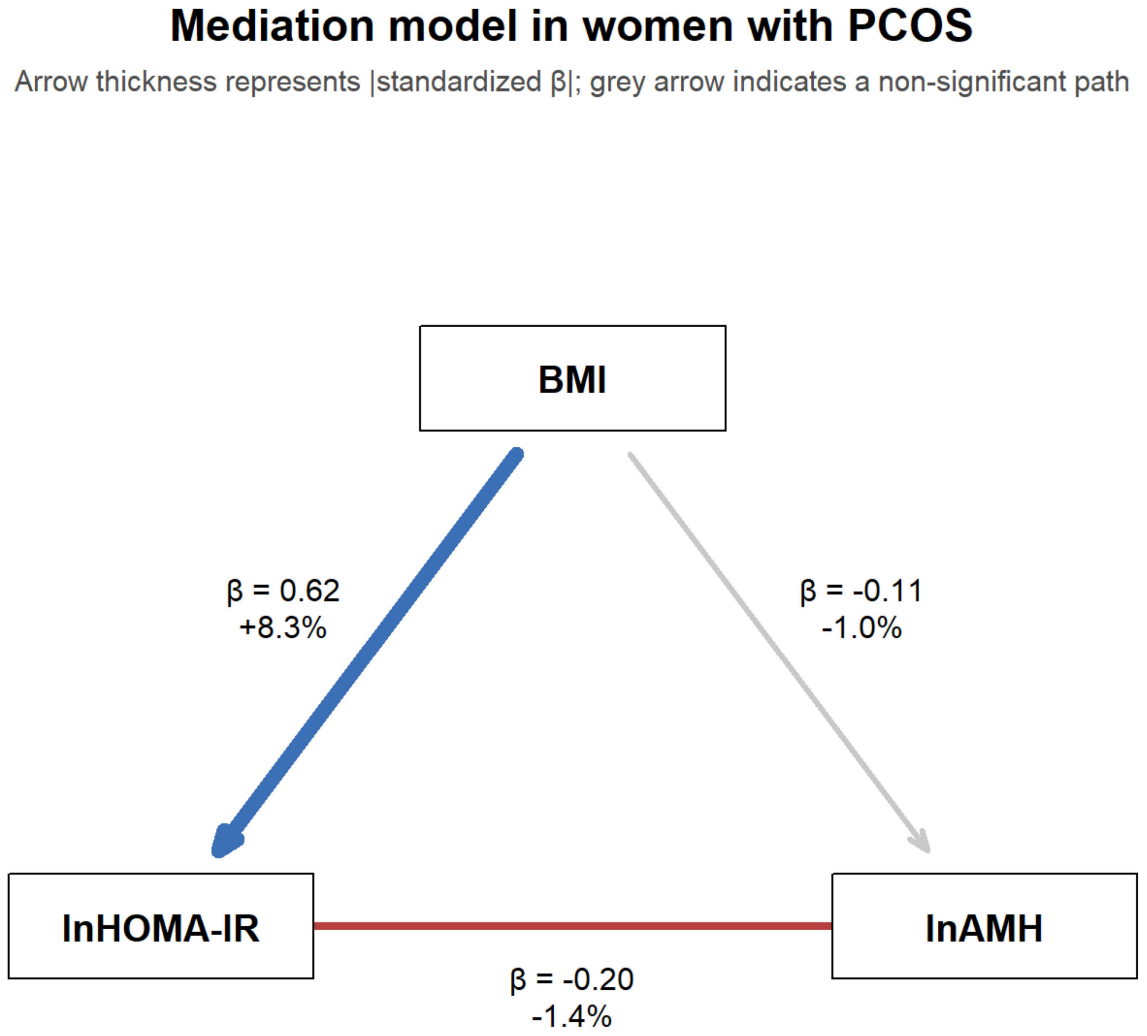
Results of the mediation analysis (generated in R).

The mediation pathway BMI → lnHOMA → lnAMH was evaluated (the corresponding path model lnHOMA→ BMI→ lnAMH did not reach overall significance*)*. The indirect effect (ACME) was −0.0116 (95% CI −0.0227 to −0.00227; p = 0.0148), while the direct effect (ADE) was −0.0101 (95% CI −0.0277 to 0.0082; p = 0.2796). The total effect of BMI on lnAMH was negative and significant: −0.0218 (95% CI −0.0349 to −0.00929; p = 0.0004). The proportion mediated was 0.53 (95% CI 0.09 to >1; p = 0.015), indicating that approximately half of the overall effect of BMI on AMH operates through lnHOMA (**Figure 3**).

On a percentage scale, a 1-unit increase in BMI was associated with an ≈8.3% higher HOMA-IR; in turn, a 10% increase in HOMA-IR was associated with an ≈1.4% lower AMH. Taken together, this corresponds to an ≈2.2% reduction in AMH per 1-unit increase in BMI. Most of this reduction reflects the indirect pathway—higher BMI elevates HOMA-IR, and elevated HOMA-IR lowers AMH—whereas the direct effect of BMI on AMH (independent of HOMA-IR) is small and not statistically significant.

## 4 Discussion

Polycystic ovary syndrome (PCOS) is a highly heterogeneous disorder, with patients presenting diverse symptoms. Consequently, therapeutic decisions must be individualized, taking into account comorbidities such as obesity, metabolic disturbances—particularly carbohydrate metabolism—and infertility. AMH levels are influenced by multiple factors, and given the increasing prevalence of fertility disorders, its role as a marker of ovarian reserve remains clinically relevant. However, previous studies have not clearly established how factors such as obesity, its distribution, or insulin resistance affect AMH concentrations. Although weight reduction has been associated with improved fertility outcomes in women with PCOS (1), the underlying mechanisms linking metabolic status and AMH remain insufficiently understood. Given the rising prevalence of obesity and its frequent coexistence with PCOS, elucidating its relationship with AMH—and similarly with insulin resistance—remains of high clinical importance (1, 10).

In this context, recent consensus discussions have proposed a re-conceptualization of PCOS as polyendocrine metabolic ovarian syndrome (PMOS), emphasizing its systemic metabolic–endocrine nature; however, this remains a proposed framework and PCOS continues to be the established terminology in current clinical and research practice (3).

Chronic low-grade inflammation is increasingly recognized as an important component of PCOS pathophysiology. Women with PCOS frequently exhibit elevated levels of inflammatory markers, such as C-reactive protein, tumor necrosis factor-α, and interleukin-6, which have been linked to insulin resistance and ovarian dysfunction (13). Recent evidence further supports this concept, showing alterations in inflammatory markers among women with PCOS and suggesting that metformin treatment may modulate inflammatory activity alongside its metabolic effects (14). Therefore, the observed association between insulin resistance and AMH in our study may partly reflect broader metabolic-inflammatory processes characteristic of PCOS. This perspective is consistent with the emerging PMOS concept, which emphasizes the systemic metabolic–endocrine nature of the syndrome (3).

Additional evidence supporting the role of metabolic dysregulation in PCOS comes from studies on adipomyokines such as irisin. A recent case–control study by Shnawa et al. demonstrated significant associations between circulating irisin levels and indices of insulin resistance and BMI in women with PCOS, further highlighting the complex interplay between metabolic status and endocrine alterations in this condition (15).

In our study, we observed a negative correlation between AMH levels and both fasting insulin and HOMA-IR. Jun et al., who studied metabolic disturbances in women with PCOS, also observed a negative correlation between AMH levels and HOMA-IR (16). Amiri et al., citing Feldman’s research and studying young women with PCOS, also confirmed that AMH levels decrease with increasing BMI and HOMA-IR (17). They suggested that measuring AMH, regardless of age or ethnicity, may be useful for assessing the risk of metabolic syndrome.

Ling-Li Tang et al. also reported a negative association between AMH levels and insulin resistance in women with PCOS; however, they did not assess the relationship between AMH and body weight (18), whereas one of the aims of our study was to evaluate the combined influence of multiple metabolic parameters on AMH levels using multivariable analysis.

In contrast, Xiang-Juan Li et al. reported a positive correlation between AMH levels and HOMA-IR, but only in women with a BMI ≥ 25 kg/m². No such association was observed in women with BMI < 25 kg/m², leading the authors to speculate on a potential role of elevated AMH in excessive insulin secretion by pancreatic β-cells (19). A positive correlation between AMH levels and HOMA-IR was also reported by Wiweko et al. (20). The divergent findings in the literature highlight the complexity of the relationship between AMH and insulin resistance in PCOS.

In our study, women with PCOS had higher BMI values compared to controls. This is consistent with published data. In the Indian population, Mohapatra et al. reported that only around 24% of women with PCOS had a normal BMI, with BMI increasing with age (21). Similarly, Pandey et al. found that while the mean BMI in healthy controls was approximately 24 kg/m², women with PCOS had a higher mean BMI of 28.28 kg/m² - and such observation is very similar to the findings obtained in our study (22).

In this study, we focused on evaluating the influence of metabolic factors on AMH levels in women of reproductive age. A negative correlation between AMH and BMI in women with PCOS had previously been observed by us, particularly in the 25–29 age group (23). Ou et al. also reported a negative association between AMH levels, BMI, and insulin resistance in the Chinese population (24). A similar negative correlation between BMI and AMH levels was observed by Güngör et al. in Turkish women with PCOS (25), as well as by Kloos et al., who reported a strong inverse association between AMH concentrations and BMI (26).

In our study, we also analyzed the interaction and mediation models involving HOMA-IR and BMI in relation to AMH levels. The data suggest that the relationship between HOMA-IR and AMH does not depend on BMI, whereas the association between BMI and AMH is less pronounced and potentially secondary to insulin resistance.

This observation is supported by findings of Anupama Bahadur et al. which show, that serum AMH is negatively correlated with HOMA-IR, while no significant correlation is observed between AMH and BMI (27). Such findings were also reported by Han Zhao et al. in the Chinese population, highlighting a negative association between AMH levels and BMI in women with PCOS. The researchers, however, suggested a positive correlation between AMH and HOMA-IR, which contrasts with the results of our study (28). This topic was also investigated by Misra et al., who observed a negative correlation between BMI and AMH, but only in women with normal body weight. They did not report any association between fasting insulin or HOMA-IR and AMH (29).

Our study did not reveal any correlation between factors such as AMH, BMI, and HOMA-IR in the control group. Similar conclusions were reached by Hamid et al., who studied the Pakistani population and Ahmed et al. in the Saudi population (30, 31). In summary, we demonstrated that fasting insulin and HOMA-IR are negatively correlated with AMH, and we also confirmed a negative correlation between BMI and AMH. Furthermore, our analysis suggests that the relationship between HOMA-IR and AMH is independent of BMI, whereas the association between BMI and AMH appears weaker and may be partly mediated by insulin resistance. Overall, these findings indicate that it may be the metabolic component (IR), rather than overall body weight (BMI), that is more strongly associated with lnAMH levels in women with PCOS.

From a clinical perspective, these observations may suggest that assessment of metabolic dysfunction in PCOS should extend beyond anthropometric measures such as BMI and include evaluation of insulin resistance, as it may represent a more relevant correlate of ovarian dysfunction reflected by AMH levels. This further supports the importance of therapeutic strategies targeting improvement of insulin sensitivity, including lifestyle interventions and pharmacological approaches, which may be particularly relevant in the management of reproductive and metabolic disturbances in PCOS. However, given the observational nature of this study, all interpretations and potential clinical implications should be made with appropriate caution and require confirmation in future prospective studies.

*Strengths and limitations:*

A strength of this study lies in its relatively large sample size and well-defined inclusion criteria. An additional strength is the use of a homogeneous Eastern European (Polish) cohort, which contributes data from a population that remains underrepresented in current literature and may improve the generalizability of metabolic–endocrine findings in PCOS.

Our study, however, has certain limitations. The relationship between AMH concentrations, HOMA-IR, and BMI is complex and may be influenced by additional factors not accounted for in this analysis. In particular, PCOS phenotypic variability may affect hormonal and metabolic profiles and should be considered in future research. Although the study includes a relatively large cohort, it is based on a single Eastern European (Polish) population, which may limit generalizability to other ethnic groups, however such a participant selection was defined as an aim of the study. Moreover, the design of the study limits the ability to infer causality between metabolic parameters and AMH levels. Additionally, lifestyle-related factors such as diet and physical activity were not systematically assessed, which may have influenced metabolic parameters.

Consequently, the results should be interpreted with caution. Future studies should incorporate phenotypic stratification and longitudinal designs to further elucidate the role of AMH in the metabolic and endocrine characterization of PCOS.

## 5 Conclusions

In this study, markers of insulin resistance, including fasting insulin and HOMA-IR, were negatively associated with AMH in women with PCOS. BMI was positively correlated with HOMA-IR and showed a weaker but significant inverse correlation with AMH in the PCOS group, whereas no significant associations between AMH and metabolic parameters were observed in the non-PCOS group. Interaction analyses did not indicate that the relationship between BMI and AMH depends on the level of insulin resistance, as the BMI × lnHOMA/lnINS interaction terms were not significant.

Mediation analysis indicated a significant indirect association, suggesting that higher BMI is associated with higher insulin resistance, which statistically co-occurs with lower AMH levels. However, no significant direct association between BMI and AMH was observed. These findings suggest thatAMH levels appear to be more strongly associated with insulin resistance than with body weight alone in PCOS.

Given the observational and cross-sectional design of the study, these relationships should be interpreted as associations rather than causal pathways. Therefore, the results should not be used to infer direct therapeutic superiority of metabolic or weight-targeted interventions, but rather to highlight the importance of metabolic assessment in this population.

Further studies incorporating PCOS phenotypic stratification and longitudinal designs are needed to better clarify the mechanisms underlying the observed associations.

## Data Availability

The original contributions presented in the study are included in the article/supplementary material. Further inquiries can be directed to the corresponding author.
